# Triple Alignment: Congruency of Perceived Preschool Classroom Social Networks Among Teachers, Children, and Researchers

**DOI:** 10.3389/fpsyg.2020.01341

**Published:** 2020-07-08

**Authors:** Jing Chen, Tzu-Jung Lin, Hui Jiang, Laura M. Justice, Kelly M. Purtell, Jessica A. R. Logan

**Affiliations:** ^1^Crane Center for Early Childhood Research and Policy, The Ohio State University, Columbus, OH, United States; ^2^Department of Educational Studies, The Ohio State University, Columbus, OH, United States; ^3^Department of Human Sciences, The Ohio State University, Columbus, OH, United States

**Keywords:** preschool social network, multiple informants, congruency, binary transformation, QAP, Jaccard index

## Abstract

Classroom social networks are influential to young children’s cognitive, social–emotional, and language development, but assessment and analyses of social networks are complex. Findings have been mixed regarding whether different informants (teachers, children, researchers) are congruent in perceiving classroom social networks. There is also a lack of discussion about the roles of network transformation (converting value networks into binary networks), a required data step for widely used statistical network analyses. This study addressed these issues based on network data of 16 preschool children containing 240 potential dyadic interactions collected from teacher ratings, child nominations, and researcher observations across 44 observation cycles over four school days. Results showed that the three informants were congruent in perceiving the classroom social network, whereas the level of congruency between the teacher-report and the researcher-report networks was the highest. Binary transformation of social networks tended to decrease the level of congruency across informants, although the level of congruency tended to be higher when more stringent binary transformation thresholds were selected.

## Introduction

Classroom social networks refer to how children connect with each other within the classroom, which have been used to infer how educational and social resources are distributed or shared among children (Moll et al., 1993; Farmer and Rodkin, 1996). Classroom social networks have gained increasing attention in the field of early childhood education since they represent children’s social experiences; are predictive of their cognitive, social–emotional, and language development; and are associated with children’s school readiness and well-being (e.g., Schaefer et al., 2010; Eggum-Wilkens et al., 2014; Delay et al., 2016).

However, the representations of classroom social networks are shaped by researchers’ decisions regarding their network assessment and analytical approaches. Common approaches to assessing classroom social networks include peer nominations (Parkhurst and Asher, 1992), teacher ratings (Lin et al., 2016), and researcher observations (Martin and Fabes, 2001). However, only a handful of studies have examined network congruency between different informants. Most of the existing studies focused on comparing teacher- and child-perceived classroom social networks in grade schools (Gest, 2006; Neal et al., 2011), whereas few were conducted in early childhood contexts or have considered how classroom social networks reported by teachers or children differ from researchers’ direct observations. Regarding network analytical approaches, although binary network transformations (i.e., converting network values from an ordinal variable into a binary variable) are required data steps for social network analyses (Handcock et al., 2016; Ripley et al., 2017), little is known about how network transformations can alter the representations of interactions between dyads and, therefore, bias the results of the congruency of networks perceived by different informants. By examining the abovementioned network assessment and analytic issues, we aimed to provide researchers with insights into better approaches to interpreting classroom social networks in the context of preschool classrooms.

### Network Assessment – Three Types of Informant Approaches

Children are insiders to the social dynamics within their social groups (Pearl et al., 2007; Neal et al., 2011). Thus, measurement of classroom social networks often relies on children as the key informants. The peer nomination method (McCandless and Marshall, 1957; Parkhurst and Asher, 1992) is a sociometric approach in which children are asked to name their peers within classrooms who fit specific social criteria, such as classmates with whom they like the most. The peer nomination approach is popular in studies focusing on children in middle childhood or adolescence due to the maturity in their language ability and social awareness (e.g., Altermatt and Pomerantz, 2005; Gest and Rodkin, 2011; Hughes and Im, 2016). When implementing the peer nomination approach in preschools, adjustments are made to ensure valid responses from young children, such as providing a classroom roster with children’s pictures to reduce the need of name recall during the nomination task, and nominations are often done in a one-on-one interview setting rather than group tests via paper–pencil questionnaires (e.g., Daniel et al., 2016). However, even with these adaptations, this child-report approach is often criticized for young children’s limited understandings of social relationships (e.g., what is friendship?) and their classmates (Hinde et al., 1985) and their higher tendency to change their responses based on their mood or immediate antecedent events compared to older children (Shin et al., 2014).

Teacher report of classroom social network is another approach that became popular in recent preschool network studies (e.g., Lin et al., 2016; Chen et al., 2018). Teachers are asked to rate the extent to which each pair of children interact with each other on a typical school day based on their observations over the past few months. Preschool teachers are thought to provide more objective information about classroom social networks based on their daily interactions with children and ongoing observations as outsider’s viewpoint (Shin et al., 2014). In addition, compared to peer nominations or researcher observations, the teacher-rating approach can be a relatively economic way to collect social network data in preschools. However, teachers’ fatigue as they try to provide ratings for every pair of children in a classroom might potentially hinder the reliability of teacher reports, especially when the classroom size increases and when multiple time points of assessment are needed. Moreover, some researchers have argued that teacher report of classroom social networks may be biased by classroom organization or management, seating arrangements, or teacher–child relationships and that teachers may overly rely on salient characteristics among children (e.g., gender, age) or overt social behaviors (e.g., hitting; Gest, 2006; Neal et al., 2011; Dawes et al., 2017).

Researchers’ direct observations of social interactions based on standardized protocols have been considered as the most objective approach to gauge classroom social networks (e.g., Martin and Fabes, 2001; Delay et al., 2016). Most of the preschool research based on direct observation was conducted during free play where researchers nonintrusively observed one child at a time using a time sampling procedure. For example, Delay et al. (2016) examined classroom social networks from 18 Head Start classrooms using a classroom observation protocol in which children were observed during free play in 10-s periods multiple times a day, several times a week across the year. Researchers were trained to live-code one child’s social interaction at a time until they went through the entire roster. This approach has been shown to predict preschool children’s academic competency (Delay et al., 2016), temperament (Neal et al., 2017), and emotionality (Fabes et al., 2012). While child and teacher reports can each provide useful perspectives on classroom social networks, direct observation of classroom social networks by trained researchers has been claimed to offer several advantages, including introducing less bias based on the characteristics of the child and allocating more attention to specific behaviors of interest (Eggum-Wilkens et al., 2014). However, researchers who used this approach tended to limit their observations within certain activities, such as free play. Compared to teachers and peers, researchers lack direct interactions with children and have less knowledge about children’s daily activities and patterns of behavior. Hence, researchers have indicated that social networks based on researchers’ direct observation might underestimate the influence of unobservable child characteristics and other classroom activities on children’s social networks (Howes et al., 1988). A brief summary of advantages and disadvantages associated with each of the three informant approaches when assessing classroom social networks is presented in Table 1.

**TABLE 1:** A summary of advantages and disadvantages of various network assessment (informants) and analysis (binary transformations) approaches based on the literature.

	**Advantages**	**Disadvantages**
**Network assessment – different informant approaches**		
Child report	It provides insiders’ perspective.	Young children might not be reliable informants and might have limited understanding of their social relationships.

Teacher rating	It is more comprehensive as it is based on teachers’ ongoing observations across classroom activities.	It can be time-consuming for teachers to rate the interaction between every pair of children. Teachers’ fatigue may reduce the reliability of their reports.
	It is an economic way for researchers to collect classroom social network data.	Teachers’ perspective can be biased by their relationships with individual children.

Researcher observation	It is considered as the most objective approach to assess classroom social networks.	Live observations are time-consuming and labor-intensive.
	It can provide more nuanced representation of classroom social networks by focusing on specific behaviors of interest.	Researcher observations are usually limited within certain classroom activities and observation windows.
		It may overlook the influence of child characteristics that are unobservable to researchers.

**Network analysis – binary transformation approaches**		
Teacher rating – selecting a cutoff on a Likert rating scale	Compared to the threshold of 1 or “rarely play,” the more stringent thresholds can filter out weak interactions.	Choosing the exact cutoff on the Likert rating scale is usually an arbitrary decision.

Researcher observations – ratio thresholds	Compared to frequency thresholds, ratio thresholds account for the potential unequal number of observations that different children receive.	Compared to frequency thresholds, ratio thresholds can reduce individual differences in children’s overall level of engagement in peer interactions.
	Compared to chance-based ratio thresholds, the 5% threshold tends to be less influenced by classroom size. (The current study is based on a single classroom, but the classroom size may play a role when multiple classrooms are included.)	The exact percentage for the fixed ratio thresholds (i.e., 5% in the current study) can be an arbitrary decision, although the literature provides some justification.
	Twice of the change threshold is more stringent than the change threshold, which allows researchers to focus more on strong or robust interactions.	Whether the fixed ration threshold (i.e., 5% in the current study) or a chance-based ratio threshold is more stringent depends on the classroom size, because classroom size is a part of the denominator when calculate the chance.

Researcher observations – frequency thresholds	This type of thresholds is straightforward in calculation.	Compared to ratio thresholds, frequency thresholds would be influenced by the unequal number of observations individual children received.
	Compared to ratio thresholds, frequency thresholds may be better in terms of retaining individual differences in their overall level of engagement in peer interactions.	The decision regarding the exact cutoff is an arbitrary decision.

Existing findings on the congruency between classroom social networks perceived by different informants are mixed. For example, Gest et al. (2003) found that peer groups identified based on a specific child-report approach called social–cognitive map were positively and reliably correlated with researchers’ direct observation in fourth- and seventh-grade classrooms (average *r* = 0.51). Howes et al. (1988), one of the few congruency studies conducted in early childhood classrooms, indicated that teacher reports and researcher observations are congruent because these two approaches identified comparable proportions of dyads in different categories of friendship relationships among children. In contrast, Cappella et al. (2012) found the teacher–child agreement on perceived classroom social network averaged only at 40% in second- to fourth-grade classrooms. Similarly, Pearl et al. (2007) found that fourth- and fifth-grade teachers seem to have difficulties in identifying social groups that are less salient (i.e., absence of desirable or undesirable social behavior) at the beginning of an academic year. Neal et al. (2011) further discussed that teacher–child agreement varied by class size, level of normative behavior, and teachers’ classroom organization in fourth-grade classrooms.

Besides the mixed findings, the existing literature tends to focus mainly on older children when examining the level of congruency across informants. This congruency issue is particularly important to be addressed in the early childhood setting for several reasons. First, preschoolers spend the majority of their school time interacting with peers, which are heavily shaped by instructional decisions made by the teachers, such as seat arrangement, grouping strategies, and conflict resolutions (e.g., Kochenderfer-Ladd and Pelletier, 2008; Gest and Rodkin, 2011). The effectiveness of these instructional strategies may rely on the extent to which teachers’ perceptions of classroom peer interactions are congruent with that perceived by children (Neal et al., 2011). Second, Gest (2006) reported that teacher–child agreement on peer group identification is higher in upper than lower elementary grades (i.e., *κ* for the combined sample of grades 3 and 5 was 0.55, while that for grade one was 0.26). However, this finding has not been well replicated and did not involve preschool classrooms. This highlights the need of this study to further understand classroom social networks in early childhood settings. Third, to our best knowledge, no research has been done to compare researchers’ observations with teacher- or child-report social networks in preschool classrooms. Triangulating with researchers’ observations, which are considered as a more objective network assessment approach, would shed light on the trustworthiness of the teacher-report approach and will improve scholarly understandings regarding the extent to which children as young as the preschool-age can be reliable informants of classroom peer interactions. To address the above gaps in the literature, the current study sought to conduct a systematic comparison among social networks perceived by the three informants in preschool classrooms.

### Network Analysis – Binary Transformation Approaches

Data of classroom social networks are often stored as matrices of binary values showing whether the social connection (i.e., a social tie) between any two children in the network is present (1) or absent (0). Alternatively, classroom social networks can be stored as matrices of ordinal values representing the frequency or quality of interaction between each pair of children. Data collected using the peer nomination approach usually contain binary values because social ties are determined by the presence or absence of children’s nominations. Data collected from teacher rating or researchers’ observations are typically in the form of value networks in which the value of a social tie equals a rating or frequency of observation. To date, the majority of existing inferential social network analytical tools handle binary networks, such as ERGM (i.e., Exponential Random Graph models; Handcock et al., 2016) and SIENA (i.e., Simulation Investigation for Empirical Network Analysis; Ripley et al., 2016). Researchers are required to transform value networks into binary networks prior to conducting these inferential social network analyses (e.g., Hunter et al., 2008; Thomas and Blitzstein, 2011; Handcock et al., 2016; Ripley et al., 2017). Even though statistical packages for value networks are available, their functions are still limited (e.g., Opsahl et al., 2010; Krivitsky and Butts, 2013; Denny et al., 2017). In addition, researchers are motivated to conduct binary transformations, which would allow them to focus more on the strong or intensive connections while reducing noise, and measurement error resulted from weak connections that might happen by chance (Thomas and Blitzstein, 2011; Siciliano, 2015).

Transforming a value network into a binary network requires the selection of a threshold. In the literature, distinct approaches have been used to transform value networks assessed via rating scales or observations. For social ties assessed via a rating scale, researchers often have to arbitrarily choose a threshold to maintain the interpretability of the findings. For example, Chen et al. (2017, 2018) studied social networks in preschool classrooms assessed by a teacher rating scale ranging from 0 (“never play”) to 4 (“always play”). They reported that 2 (“sometimes play”) was chosen as the cutoff value for the binary transformation because it represented the grand mean frequency of interactions among children across classrooms. Siciliano (2015) studied advice networks among teachers. The frequency at which one teacher sought advice from another ranged from 1 (never) to 5 (daily). Siciliano chose 3 (monthly) as the threshold for the binary network transformation in order to eliminate weak ties, or infrequent interactions, which were not effective in explaining how knowledge flowed within organizations.

For social ties measured by observational frequencies, the threshold for binary transformations documented in the literature can be summarized into two types: ratio thresholds and frequency thresholds. For ratio thresholds (Schaefer et al., 2010; Daniel et al., 2013), researchers calculate a ratio – dividing the frequency of interaction between child *i* and child *j* by the frequency of interaction between child *i* and any classmates. Then, researchers compare this ratio with the baseline, which is operationally defined as 1/(effective classroom size – 1), representing the situation where the frequency of interactions could happen by chance. Schaefer et al. (2010) proposed that if the ratio is at or above the baseline, the interaction from child *i* to child *j* is coded as one; otherwise, it is coded as zero; Daniel et al. (2013) proposed a more stringent approach that the threshold is at or above twice the baseline. Alternatively, in the context of global telecommunications, Monge and Matei (2004) proposed that the baseline ratio can be meaningfully set at 5%. They argue that individuals tend to spend the majority of their time and effort interacting with only a few others regardless of the number of members in a network. These three ratio thresholds (i.e., chance level, twice the chance level, 5% level) allow the binary transformation process to vary by individuals’ levels of engagement in peer interactions and can account for the potential unequal number of observations individual kids receive (Schaefer et al., 2010). As an alternative to the use of ratio thresholds, Cugmas et al. (2019) applied a frequency threshold. They studied the formation of networks among preschool children and specified that the frequency of observed interactions between pairs of children should be coded as one when it was above half of the median of the interaction frequencies among all possible pairs in the network.

With different binary transformation approaches presented above and summarized in Table 1, surprisingly there is a lack of close examination into how these different forms of network representations can potentially bias our understanding of classroom social networks. The current study addressed this issue from the perspective of congruency across informants with or without different types of binary transformations.

In all, with regard to the research gaps related to network assessment (i.e., three types of informants) and network analysis approaches (i.e., different types of binary transformation approaches), two specific research questions were addressed in this study: (1) To what extent are classroom social networks as perceived/reported by children, teachers, and researchers congruent with one another? (2) To what extent would binary network transformations affect the level of congruency among informants’ perceived classroom social networks? The levels of congruency across informants were compared based on the original network data, as well as binary network data transformed by each of the approaches presented above.

## Materials and Methods

### Participants

The participants of this study were 16 preschool-aged children enrolled in one preschool classroom in a nonprofit early childhood center located in a Midwestern city of the United States. This early childhood center, which operates on a ten-hour day (7:30 AM–5:30 PM), provides comprehensive, continuous services to children from diverse backgrounds with various funding streams at the local, state, and federal levels. The classroom was instructed by a three-teacher team: a master teacher and two lead teachers. All of the teachers in the classroom had an associate’s degree or higher in a relevant field (e.g., early childhood education) and 2–5 years of teaching experience in preschool. The class contained 20 children, 16 of whom were consented (six girls and ten boys) to participate in the study. The nonconsented children did not participate in the data collection process. Their social network information provided by peers was excluded from the data analysis.

The 16 consented children formed a maximum of 240 dyads, which was the unit of analysis of this examination. All children were enrolled in the 5-day full-day program. The classroom was a mixed-age classroom containing children from ages 3–5. The average age in months was 46.19 (*SD* = 8.19, range = 35–58). Using administrative records, 11 children were identified as black or African American, four were identified as white, and one child as other; 33% children resided in home where the highest level of maternal education was high school, 7% had some college experience without a degree, 27% had a bachelor’s degree, and 33% had a master degree or higher.

### Measures

The classroom social network was assessed in the fall of the year (September) using three approaches: peer nomination, teacher rating, and researcher observations.

#### Peer-Report Classroom Social Network

In one-on-one interviews with research staff, children were provided a classroom roster with pictures of all classmates and were asked to nominate with whom they liked to play the most in a one-on-one interview (Daniel et al., 2016). Daniel et al. argued that this interview approach with pictures of classmates could help children stay on-task during the nominations and could help them overcome memory or language barriers. Children were allowed to nominate as many peers as they wanted but were encouraged not to nominate everyone in the classroom. Each interview lasted less than 5 min.

#### Teacher-Report Classroom Social Network

The master teacher rated the extent to which every pair of children in the classroom played with each other on a typical school day on a 5-point Likert scale (0 = never, 4 = always) based on her observation during the past 3 months (Lin et al., 2016). Examples of play interactions provided to the teacher included pretend play, giving and sharing toys or ideas, playing with balls on the playground, collaborating on class projects, and reading books together.

#### Researcher-Observed Classroom Social Network

Adapted from Martin and Fabes (2001), trained researchers coded the frequency at which each child played with peer(s) in the forms of cooperation/discussion (children played or worked together with one or more peers on the same activity reciprocally and constructively, such as building a block tower together or running around and pretending to be superheroes), parallel play (children played next to but not with peers on the same activity), child-led activity (children participated in or paid attention to a child-led activity), rough and tumble play (children playing together physically and roughly, such as chasing, wrestling), victimization (children were the physical or verbal victims of other children), aggression (children threw, hit, or verbally attack other children), ignored (children made an unsuccessful attempt to interact with others), and no interaction (children were engaged in solitary activities, observing others, or unoccupied). For the purpose of the current study, researcher-observed classroom social networks were formed based on the frequencies of cooperation/discussion between pairs of children. This is because compared to other types of peer interactions, it matches better with the nature of interactions assessed via play-most peer nomination and teacher ratings of play interactions between peers.

Researchers conducted observations in five consecutive school days. Each day, there was one 2 h observation window in the morning and another 2 h observation window in the afternoon. In each observation window, a researcher observed one child at a time for 30 s and coded the social interaction and with whom that the child interacted during the next 30 s. The researchers then moved to the next child on the classroom roster until all the children were observed, which was considered a complete observation cycle. The researchers then started the next observation cycle until the end of the 2 h window. On average, each child was available to be observed 72% (*SD* = 0.31) out of a total of 44 coding cycles, which was 33.07 times (*SD* = 10.76). Children were not available to be observed when they were absent, in the bathroom, or asleep.

To test the validity of the three classroom social network measures, we associated these network measures with children’s school records of language ability, social and emotional development, and self-regulation ability, which have been shown in the literature positively related with the amount of play interactions preschoolers engage in classrooms (e.g., Mashburn et al., 2009; Justice et al., 2011; Ribeiro et al., 2017; Chen et al., 2018). To account for the small sample size, the 95% confidence intervals (CIs) of correlations coefficients were calculated using a bootstrap approach with 1,000 times of resampling. For the child-report network assessed by the peer nomination measure, the total number of play-most nominations a child received from the classmates was associated with children’s social and emotional development (*r* = 0.74, CI = [0.29, 0.89]) and self-regulation (*r* = 0.65, CI = [0.19, 0.86]). Its correlation with language ability was not significant (*r* = 0.38, CI = [−0.12, 0.87]). Based on the teacher-report network, the total score of teacher ratings associated with each child was significantly correlated with children’s language ability (*r* = 0.65, CI = [0.29, 0.94]), social and emotional development (*r* = 0.53, CI = [0.30, 0.76]), and self-regulation skills (*r* = 0.39, CI = [0.02, 0.69]). Regarding the researcher-report network, the total number of peer interactions associated with a child was also significantly correlated with language ability (*r* = 0.65, CI = [0.29, 0.93]), social and emotional development (*r* = 0.66, CI = [0.34, 0.85]), and self-regulation skills (*r* = 0.56, CI = [0.18, 0.79]). Hence, the validity of each of the three network assessment approaches was supported in the current study.

### Analytical Approach

**FIGURE 1:**
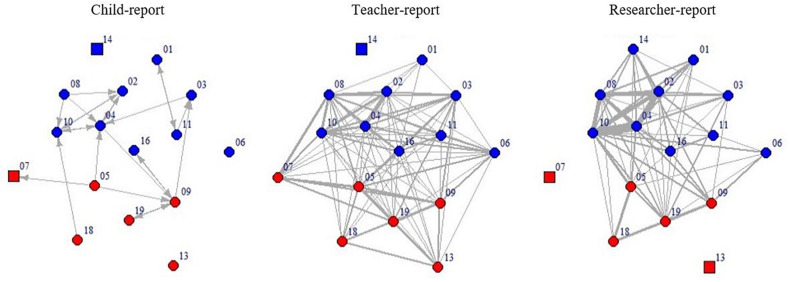
Classroom social network graphs based on child nominations, teacher ratings, and researchers’ observations. Node color represents gender (blue = boy, red = girl); node shape represents whether the child had available information for the particular assessment (circle = yes, square = no); the thickness of edges represents the intensity of peer interactions; the numbers besides notes represent child IDs. The positions of nodes are identical across plots. For the child-report network, the arrows are pointed toward the nominees.

Three sets of social network data were created based on the reports from children, teachers, and researchers. As explained in a previous section, the original network data obtained from the three types of informants were in different formats: a binary network from peer nominations indicating whether or not one child nominated the other in each dyad, a value network from teacher ratings indicating the intensity of play interactions between pairs of children, and a value network from researchers’ observations representing the frequency at which pairs of children interacted throughout 44 observation cycles. All of the data were converted to a 16 × 16 matrix, where the rows and columns represented the 16 children in an identical order. The cell [*i*, *j*] in the matrix represented peer interactions between pairs of children. Figure 1 presents the classroom social network graphs based on the original child report, teacher rating, and researchers’ observations.

To address the first research question targeted on the network assessment issue, namely, the extent to which the classroom social networks perceived by children, the teacher, and researchers are congruent with each other, we tested network associations based on original values (hereafter called original networks) using the Quadratic Assignment Procedure test (QAP, Krackardt, 1987). The QAP test is a graph correlation analysis, which applies a hypothesis testing approach to examine the correlation between two networks while taking into account possible interdependency among dyads via a Monte Carlo simulation approach, which can be applied to both valued and binary networks.

The second research question focuses on network congruency across the three types of informants after binary transformations. The child-report network did not need a binary transformation as it was already in the binary format. The transformation approaches for the teacher- and the researcher-reported networks were selected, respectively, based on the literature reviewed in the previous section. The original teacher-report network was binary transformed based on three thresholds: 1 (“rarely play”), 2 (“sometimes play”), and 3 (“often play”). The grand-mean teacher rating was 1.35. Hence, the threshold of 1 was considered the least stringent criterion, and the threshold of 3 was considered the most stringent criterion. The rating of 4 (“always play”) was not tried as a threshold because it occurred only twice among all the possible interactions.

The original researcher-report network was binary transformed, where three ratio thresholds and three frequency thresholds were tested. As explained in the literature review, ratio thresholds referred to the reference point for the ratio of a child’s interaction frequency with a peer divided by the child’s total interaction frequency with all classmates. The interaction from this child to the peer was coded as one if the ratio was at or above a certain ratio threshold. The three ratio thresholds included (1) Schaefer et al. (2010), where the ratio threshold is the chance level [i.e., 1/(effective classroom size - 1) or 6.7% based on the current study]; (2) Daniel et al. (2013), where the ratio threshold is twice the chance level (i.e., 13.4% based on the current study); and (3) Monge and Matei’s (2004) approach, where the ratio threshold is 5%.

The frequency thresholds were identified directly based on interaction frequencies, which were identical across children regardless of their overall level of engagement in peer interactions. First, interactions between a pair of children were coded as one if the interaction frequency between them was greater than half of the median of the interaction frequencies among all possible pairs in the network (Cugmas et al., 2019). In this study, the median value was 1, which suggested that the interaction between pairs of children was coded as 1 if they were observed interacting with each other at least once. Then, more stringent frequency thresholds were explored based on the distribution of the observed interaction frequencies, including the 75th percentile and the 90th percentile, which corresponded to the interaction frequency of 2 and 4, respectively.

To examine the congruency between pairs of binary networks, we applied two approaches to triangulate the results – the QAP procedure as explained above and the Jaccard indices (Real and Vargas, 1996). Jaccard index is exclusively used for binary networks and has been widely used in the literature to represent the overlap between networks (e.g., Ripley et al., 2017; Vörös and Snijders, 2017; Daniel et al., 2019). A Jaccard index is calculated by the proportion of common attributes shared between two networks (i.e., *J* = C/(*A* + *B* + *C*), where *C* is the number of dyads that are identical in both networks, and *A* and *B* are the numbers of dyads that are uniquely identified in either network). The same as the QAP coefficient, the Jaccard index ranges from 0 to 1; a higher value indicates greater similarity between two networks.

## Results

### Preliminary Analyses

Based on the child-report classroom social network, 11% of all the potential social ties (*N* = 240) were present, each representing a child nominating one peer as someone he/she liked to play with the most. Based on the teacher rating, the average frequency of interaction between pairs of children was 1.35 (*SD* = 0.90) on the scale ranging from 0 (never play) to 4 (always play). Based on researchers’ observations across the 5-day observation window, on average a pair of children tended to play with each other 1.47 times on average (*SD* = 2.65, range = 0–20). We calculated the proportions of present social ties from each of the binary transformed classroom social networks. Three of the networks were transformed from teacher-report networks, with the thresholds set at 1, 2, and 3. The proportion of present social ties were 85, 38, and 11%, respectively. For researcher-report social networks with the ratio the thresholds set as the chance, twice of chance, and 5%, the proportion of present social ties was 32, 19, and 41%, respectively; with the frequency thresholds settings as the frequency of 1, 2, and 4, the proportion of present social ties was 53, 32, and 10%, respectively.

### Network Assessment Issue – Congruency Across Informants

To answer the first research question, the QAP test (Table 2) showed that the correlation between child-report and teacher-report networks (*r* = 0.33, *p* < 0.001), that between child-report and researcher-observed networks (*r* = 0.50, *p* < 0.001), and that between teacher-report and researcher-observed networks (*r* = 0.57, *p* < 0.001) were all statistically significant.

**TABLE 2:** Graph correlations (QAP tests) between pairs of original networks.

	**1**	**2**	**3**
Child-report network (whether or not one child nominated the other)	–		
Teacher-report network (0 = never play, 4 = always play)	0.33***	–	
Researcher-report network (observed frequency of play interactions)	0.50***	0.57***	–

***p < 0.001.

### Network Analyses Issue – Influence of Binary Transformations

To answer the second research question, the results of QAP tests are presented in Table 3. First, the correlations between child- and teacher-report networks increased from 0.18 (*p* = 0.055) to 0.52 (*p* < 0.001) as the threshold of the teacher report increased from 1 (“rarely play”) to 3 (“often play”). However, the level of congruency decreased compared with the situation when no binary transformation was conducted (*r* = 0.33, *p* < 0.001), except when the most stringent binary cutoff was applied to the teacher-reported network. Second, the levels of congruency between child- and researcher-report networks transformed based on the three ratio thresholds were relatively stable (*r*’s = 0.22–0.26, *p*’s = 0.003–0.008). In contrast, with frequency thresholds, the correlation between child-report and researcher-report networks increased from 0.14 (n.s.) to 0.44 (*p* < 0.001) as the frequency threshold increased from 1 to 4. However, with any type of binary transformation threshold, the level of congruency between child- and researcher-report networks was lower than that between the two original networks (*r* = 0.50, *p* < 0.001). Third, the correlations between teacher- and researcher-report networks appeared to be stronger when the more stringent binary transformation thresholds were chosen for both informants. Specifically, the correlation was highest (*r* = 0.63, *p* < 0.001) when the teacher-report network was transformed based on the threshold of 3 (“often play”), and the researcher-report network was transformed with the frequency threshold of 4 (the 90th percentile frequency). Although the rest of the situations (when less stringent thresholds were applied to networks based on either informant) yielded relatively weaker correlations especially compared with the situation when no binary transformation was conducted (*r* = 0.57, *p* < 0.001), these coefficients were all statistically significant. The only exception was the correlation between the teacher-report network with the threshold of 1 (“rarely play”) and the most strictly defined researcher-report network with the frequency threshold of 4 (the 90th percentile frequency).

**TABLE 3:** Graph correlations (QAP tests) between pairs of binary networks.

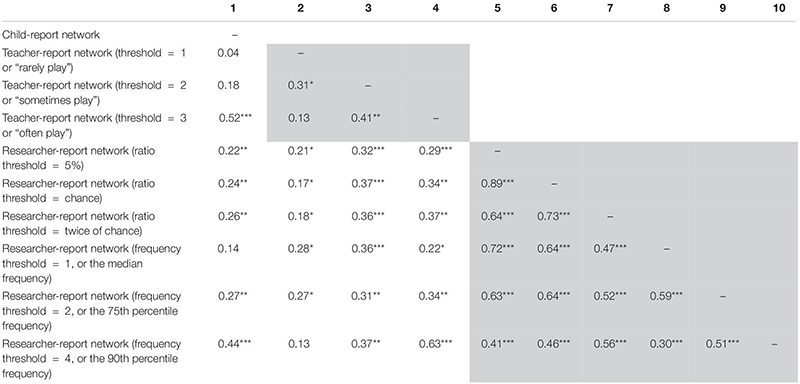

The gray areas are graph correlations between pairs of binary networks from the same informants. ***p < 0.001, **p < 0.01, *p < 0.05.

The Jaccard indices indicating proportions of overlap between pairs of binary networks are presented in Table 4. As mentioned above, these analyses were conducted to triangulate with the results of the QAP tests. In line with QAP results, Jaccard indices showed that, first, the level of congruency was generally higher between the teacher- and the researcher-report networks when compared with the child-report network; second, the level of congruency tended to increase as the binary threshold for the teacher-report network changed from 1 (“rarely play”) to 3 (“often play”) and as the frequency threshold for the researcher-report network changed from the median (1) to the 90th percentile (4), and third, when the researcher-report network was transformed with different ratio thresholds, the levels of congruency between the child- and the researcher-report networks were stable but only at the moderate level (i.e., 17–21% of overlapped social ties). However, contrary to the results of the QAP tests, the least stringent thresholds yielded a high level of congruency between the teacher- and researcher-report networks, in which the proportion of overlapped social ties was 61%.

**TABLE 4:** Proportions of overlap (Jaccard indices) between pairs of binary networks.

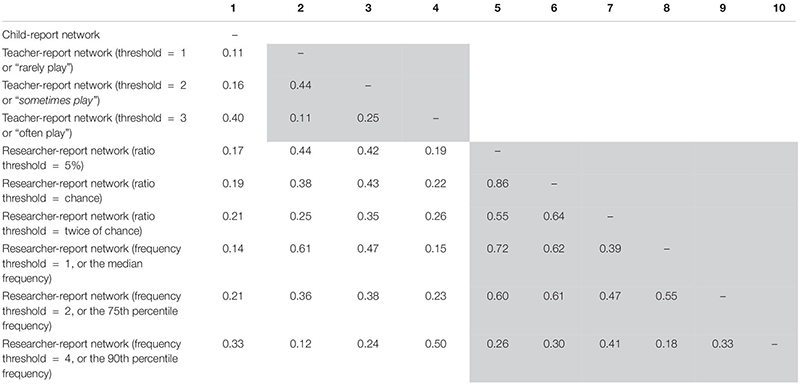

*The gray areas are proportions of overlap between pairs of binary networks from the same informants.*

## Discussion

In this study, we examined how network assessment (based on different informants) and network analysis (based on different network transformation methods) influenced our understanding of classroom social networks. From the perspective of network assessment, existing measures of classroom social networks rely heavily on different informants’ observations. Surprisingly, to the best of our knowledge, no single study has systematically examined the level of congruency across children’s, teachers’, and researchers’ perceived social networks. The issue of congruency can be particularly important in the preschool context, where the validity of young children’s first-person perspective can be a major concern. From the perspective of network analysis, despite the common practice of binary network transformation in social network studies, selecting a binary transformation threshold seems to be an arbitrary decision for many researchers. Prior to the current study, there was no available information on how different thresholds for binary network transformation could potentially influence network representations. The current study contributes to the field by rigorously examining the congruency of classroom social network perceived by preschoolers, teachers, and researchers and comparing the level of congruency across informants via different types of binary network transformation approaches.

Overall, the perceived social networks by children, teacher, and researchers in the preschool context were congruent with each other. This is supported by the visual representations (Figure 1) and further quantified by the QAP correlations based on both the original and binary transformed networks (Tables 2, 3). The finding suggests that preschool children are reliable informants of their classroom social networks. However, the level of congruency was higher between the teacher- and researcher-report networks than their congruency with the child-report network. This conclusion holds both when the congruency was examined based on the original networks and when based on transformed networks. It is possible that teachers and researchers are outsiders of peer interactions among children, both of whom may rely on common observable cues to identify peer interactions. Comparatively, children, who are insiders of the networks, may perceive their peer interactions beyond the observable cues (e.g., level of trust), which can make their perceptions qualitatively different from the other informants (Pearl et al., 2007; Neal et al., 2011). Another methodological explanation is that the child-report network contains information about the directionality of peer interactions (e.g., who likes to play with a child the most), whereas both the teacher- and the researcher-report networks represented peer interactions between pairs of children without specifying the directionality (i.e., who initiated the interactions) based on the assessment approaches applied in the field (e.g., Martin and Fabes, 2001; Schaefer et al., 2010; Chen et al., 2018; Lin et al., 2019).

Unsurprisingly, the level of congruency among child-, teacher-, and researcher-report networks generally decreased as certain binary network transformation approach was applied. It can be explained by the literature suggesting that binary network transformations tend to reduce information (e.g., Thomas and Blitzstein, 2011; Pilny and Atouba, 2018). This is why researchers have been developing analytical approaches to handle value networks without transformation (e.g., Opsahl et al., 2010; Krivitsky and Butts, 2013; Denny et al., 2017).

Although binary network transformation approaches tend to decrease the level of congruency across informants, the level of congruency can be higher when more stringent thresholds were selected for network transformation. In the current study, for the teacher-report network, the stringent threshold was 3 (“often play”); for the researcher-report network, the stringent ratio threshold was the twice-of-chance threshold, and the stringent frequency threshold was the frequency of 4 (the 90th percentile). With these three stringent thresholds, the proportions of present social ties were 11, 19, and 10%, respectively. We thus conjecture that the higher congruency level across the informants based on the stringent thresholds could be because the stringent thresholds filtered unreliable observations of peer interactions and retained more robust peer interactions across all informants. Another potential explanation is the selective nature of the child-report network as we encouraged children to consciously nominate peers whom they liked to play with the most. However, researchers should be mindful of the tradeoff when choosing to use the stringent threshold. Although the stringent threshold may yield greater congruency across informants, it can oversimplify the complex nature of social networks within classrooms.

For the researcher-report network, the levels of congruency across informants were only moderate (*r*’s = 0.17–0.37) with ratio thresholds, whereas higher levels of congruency were found with certain frequency thresholds (*r*’s = 0.14–0.63). It is possible that the ratio threshold approaches might have canceled out the variation of children’s overall level of engagement in peer interactions, which is a piece of essential information for classroom network research. In the current study, for instance, Tom (child 1 in Figure 1) was observed to interact with peers 12 times in total, and Bob (child 2 in Figure 1) was observed to interact with peers 44 times in total. Tom tended to interact with a lot of peers at a lower frequency level, whereas Bob tended to interact with a few peers very frequently. As a result, with the 5% binary threshold proposed by Monge and Matei (2004), Bob’s network connections might be fewer than Tom’s, while the raw interaction frequencies would suggest the opposite pattern. Although Schaefer et al. (2010) suggested that the ratio thresholds can account for the influence of unequal number of observations received by different children due to their absence, it could hinder the congruency across informants by canceling out individual differences in children’s overall level of engagement in peer interactions.

In the current study, we applied both the QAP tests and the Jaccard index to examine network congruency. The two network correlation approaches resulted in a major inconsistent finding. With the QAP tests, the highest level of congruency between teacher- and researcher-perceived networks occurred when the most stringent threshold was applied. Surprisingly, with the Jaccard index, the highest level of congruency occurred when the least stringent threshold was applied. Note that the Jaccard index is a widely used approach to represent the proportion of overlap between pairs of binary networks (Real and Vargas, 1996). However, the proportion of overlap is contingent upon the number of present social ties available in the networks. Mathematically, when the least stringent threshold is applied [e.g., 1 (“rarely play”) for the teacher-report network and 1 (the median) for the researcher-report network], the large number of present social ties available in the two networks naturally increases the likelihood that the two binary networks will overlap on their social ties. Our findings, therefore, suggest that the Jaccard index might be biased toward networks with greater network density or with a higher proportion of present social ties in the networks. Researchers need to take into account the proportion of present social ties in the networks when interpreting Jaccard scores.

There are a few limitations in the current study. First, this study was conducted in a single preschool classroom of 16 consented children who formed 240 dyadic social ties. We focused on one classroom in part because the data collection process was intensive, and strict protocols must be followed to ensure we have valid representations of the classroom social network from the perspectives of children, teachers, and researchers. However, the small number of children in the single classroom may limit the generalizability of the findings. We acknowledge the trade-off between meeting scientific standards and generalizability. Future study is needed to replicate the findings in other classroom settings with more diverse student populations.

Second, we chose peer nomination as the approach to collect children’s perspective of classroom social network, but alternative child report approaches can be considered in the future. For example, Parker and Asher (1993) applied a roster-and-rating approach asking children to rate the extent to which they like to play with each classmate on a Likert scale; Dishion and Tipsord (2011) applied a social–cognitive map procedure, which requires children to identify dyads of classmates who like to play together, and Daniel et al. (2016) administered a paired comparison task, in which children are presented with pictures of pairs of classmates and are asked to choose one over another in each pair in terms of whom they like to play with better. There are pros and cons with each option. Although the roster-and-rating scale forces children to consciously consider their interactions with every classmate, the social–cognitive map procedure can better handle children’s absence, and the paired comparison task differentiates the closeness of interactions associated with each child, we chose the peer nomination task because we believe it is relatively more straightforward and age-appropriate for preschool children given the purpose of the current study.

Third, the current study mainly focuses on informants’ congruency in a specific type of classroom social network that focuses on play interactions. Network congruency may differ when we examine negative peer interactions, such as aggression and victimization. We chose to focus exclusively on play interactions because traditionally it has been suggested that child reports on peers’ negative behaviors tend to be less reliable than their reports on positive behaviors (Cohen and Van Tassel, 1978; Asher et al., 1979). Asking children to report peers’ negative behaviors may also encounter an ethical problem, as it might prompt children to judge their peers negatively (Hymel and Asher, 1977). However, there is a growing interest in classroom social networks based on negative social interactions (e.g., Ahn et al., 2013; Huitsing et al., 2019) given the prevalence of aggression or school violence. Particularly, studies have suggested that physical aggression is especially common during early childhood than other life stages, although it is unusual for young children to physically harm others seriously (e.g., Tremblay et al., 2004; Alink et al., 2006). Thus, future studies may take into account both positive and negative peer interactions when examining classroom social networks, which will help to form a more comprehensive understanding of the congruency of perceptions from different informants.

Despite the limitations, this is a pioneering study that simultaneously examined the congruency of classroom social networks perceived by children, teachers, and researchers in the preschool contexts and an innovative research that compared multiple types of binary network transformation approaches on the congruency across informants. Our findings suggested that while preschoolers, teachers, and researchers are generally congruent in their perception of classroom social networks, the level of congruency is higher between the perception of teachers and researchers. This systematic comparison between the three informant approaches sheds light on the pros and cons of choosing each approach for assessing preschool classroom social networks. Another key practical contribution of the study lies in the finding that binary transformation, a common network analysis procedure, can decrease the level of congruency across informants, except when stringent binary transformation approaches are applied. This finding cautions network researchers when different informant assessment and binary transformation approaches are used. Overall, this study has great methodological and practical implications for researchers as they consider different network assessments and transformation approaches to study the dynamic social networks in preschool classrooms.

## Data Availability Statement

The datasets generated for this study are available on request to the corresponding author.

## Ethics Statement

The studies involving human participants were reviewed and approved by the Ohio State University. Written informed consent to participate in this study was provided by the participants’ legal guardian/next of kin.

## Author Contributions

JC conceptualized this study, organized the data, conducted the analysis, and wrote the first draft of the manuscript. T-JL conceptualized this study and revised the manuscript. HJ, LJ, and KP provided critical review of the manuscript. T-JL, LJ, KP, and JL acquired financial support for the project leading to this publication. All authors contributed to the article and approved the submitted version.

## Conflict of Interest

The authors declare that the research was conducted in the absence of any commercial or financial relationships that could be construed as a potential conflict of interest.
